# Effectiveness of stigma reduction interventions and outbreak response adaptations in infectious disease outbreaks: a systematic review

**DOI:** 10.3389/fpubh.2026.1755092

**Published:** 2026-02-03

**Authors:** Amy Paterson, Ruan Spies, Chambrez-Zita Zauchenberger, Ashleigh Cheyne, Piero L. Olliaro, Amanda Rojek

**Affiliations:** 1 Pandemic Sciences Institute, University of Oxford, Oxford, United Kingdom; 2 Oxford University Clinical Research Unit, Ho Chi Minh City, Vietnam

**Keywords:** discrimination, outbreak, pandemic preparedness, public health and social measures, stigma

## Abstract

**Introduction:**

Stigma is a common and recurring feature of infectious disease outbreaks where it may have detrimental effects on individual wellbeing and undermine outbreak response. This systematic review explores stigma reduction interventions in infectious disease outbreaks.

**Methods:**

Eligible studies were searched for in Medline, Embase, PsycINFO, and Global Health databases and through reference screening. Risk of bias was assessed using study design-specific tools and the results of included studies underwent narrative synthesis.

**Results:**

Eleven studies conducted across coronavirus disease 2019 (COVID-19), Ebola disease, mpox, severe acute respiratory syndrome (SARS), and a hypothetical infectious-disease scenario, met the inclusion criteria. Five studies reported reductions in stigma, four reported mixed or null results, and two reported increases in stigma. The most promising strategies for outbreak-related stigma reduction were embedding anti-stigma messaging within health communication, providing psychosocial support, and fostering genuinely participatory community involvement.

**Discussion:**

Evidence on how to effectively reduce stigma during outbreaks remains limited. Strengthening the theoretical foundations, measurement tools, and evaluation designs of stigma-reduction interventions will be essential to inform evidence-based outbreak preparedness and response policies. This would help decision-makers ensure that risk communication, community engagement, and service delivery minimise stigma and improve uptake of testing, care, and preventive measures.

## 1 Introduction

Stigma is a common and recurring feature of infectious disease outbreaks. It can be defined as the social devaluation and exclusion of individuals or groups based on perceived association with a discrediting illness (1, 2). The sources of outbreak-related stigma are multifaceted. It often stems from fear and uncertainty surrounding a novel disease or moralisation of the illness, resulting in blame being placed on certain individuals or groups for disease transmission (1). Outbreak control measures, such as surveillance or quarantine, can also inadvertently reinforce stigma, particularly when they are intrusive or restrict personal freedoms (1). Similarly, public health messaging that is alarmist or over-emphasises preventability through basic hygiene may exacerbate stigma.

The detrimental impact of stigma on both physical and psychosocial wellbeing is well-described (3). Individuals stigmatised during outbreaks may experience psychological distress, depression, and a reluctance to seek medical care, thereby compounding their vulnerability (4–6). Furthermore, stigma may undermine critical public health measures by discouraging community cooperation with outbreak response efforts, fostering distrust towards health authorities, and lowering adherence to preventive behaviours (1).

Existing systematic reviews have primarily examined stigma interventions in established infectious diseases such as HIV, tuberculosis, or leprosy. However, outbreaks present distinct challenges for stigma mitigation. These events tend to arise suddenly, progress rapidly, and occur within diverse sociocultural settings, complicating the design, implementation, and evaluation of interventions (7, 8). While these distinctions limit the transferability of evidence from non-outbreak infectious disease contexts, commonalities in stigma across outbreaks points to the potential for adaptable, cross-outbreak learnings (9).

Although the importance of addressing stigma during outbreaks is increasingly recognised, evidence on how best to reduce it remains limited. A clearer understanding of which outbreak-specific approaches have been evaluated, and with what results, is therefore critical to strengthening preparedness and response.

This systematic review identifies and synthesises evidence from studies evaluating outbreak-related stigma reduction interventions. By mapping the available evidence and assessing the effectiveness and methodological quality of these studies, the review aims to inform public health preparedness strategies and support the development of stigma-sensitive approaches to outbreak response.

## 2 Methods

The protocol for this review was prospectively registered with the International Prospective Register of Systematic Reviews (PROSPERO) (registration number CRD420251007553). The review has been reported in accordance with the 2020 Preferred Reporting Items for Systematic Reviews and Meta-Analyses (PRISMA) guidelines (10).

### 2.1 Eligibility criteria and search strategy

Studies were deemed eligible for inclusion in this systematic review if they fulfilled all the inclusion criteria and none of the exclusion criteria outlined in Table 1.

**Table 1:** Eligibility criteria for study inclusion in systematic review.

Category	Inclusion criteria	Exclusion criteria
Population	Studies that include participants of any age, gender, or geographic location who are affected by, endorse, enact, or are positioned to influence stigma associated with an infectious disease outbreak. These may be individuals with or without personal experience of the disease.	Studies focused on non-outbreak disease contexts. Studies focused on marginalised groups being stigmatised during the time period of an outbreak but not due to association with the disease.
Intervention	Studies that describe the implementation of at least one intervention (including adaptations to policies and procedures) intended to reduce stigma associated with an outbreak-prone infectious disease.	Studies that describe general stigma reduction measures not applied to the context of infectious disease outbreaks.
Comparison	Studies that include a description of the evaluation of the interventions in comparison to routine practice, alternative interventions, or no intervention.	Studies without clear evaluation of effectiveness of interventions.
Outcome	Studies that report the effectiveness of interventions in reducing any form of outbreak-related stigma or improving social acceptance.	Studies that do not provide relevant data on intervention impact on stigma.
Study design	Peer-reviewed studies with comparative/evaluative design including: - Randomised controlled trials - Quasi-experimental studies - Observational studies with comparative design - Mixed-methods studies - Qualitative studies	Studies without a comparative or evaluative component; studies relying solely on conceptual discussions or theoretical models; protocols, guidelines, book sections, case reports, opinion pieces (editorials, viewpoints, commentaries); conference abstracts; preprints and other non-peer-reviewed literature.

Effectiveness was defined broadly, reflecting heterogeneity in stigma constructs and outcome measures across studies (11). An intervention was considered effective if it demonstrated improvement in one or more stigma-related domains, including reductions in stigma scores, positive changes in stigma-related attitudes or beliefs, and/or improvements in social acceptance or reintegration-related outcomes. No single threshold or composite definition was imposed. Instead, effectiveness was assessed relative to each study’s stated outcomes and synthesised narratively.

Both quantitative and qualitative studies were considered informative for assessing intervention effects. Quantitative studies contributed evidence of effect through measured changes in stigma-related outcomes, while qualitative studies contributed evidence of effect through participant-reported changes in stigma, social acceptance, or experiences of discrimination following intervention implementation. Qualitative findings were synthesised alongside quantitative results to capture effects that were not amenable to standardised measurement and to reflect the social and contextual nature of stigma in outbreak settings.

A search strategy was developed in collaboration with a university librarian. Search terms relating to ‘stigma or social acceptance’ and ‘infectious disease outbreaks’ were combined using Boolean operators to search keyword fields and indexed subject headings. To ensure inclusion of relevant infectious disease outbreaks, the following lists were reviewed: the World Health Organization (WHO) list of priority diseases, the UK Government’s list of High Consequence Infectious Diseases, WHO outbreak news reports, and the United States’ Centers for Disease Control and Prevention (CDC) list of international outbreaks. Any disease appearing on more than one list was included in the search terms.

Searches of Medline, Embase, PsycINFO, and Global Health were conducted without restrictions on language or publication date. Full search strategies for each database are presented in the supplementary material. Records published through to 11 March 2025 were retrieved. Reference lists of included studies identified through the electronic search were also reviewed.

### 2.2 Study selection

All citations retrieved from the search were uploaded to Rayyan systematic review software and deduplicated (12). Three independent reviewers screened a random 10% of titles and abstracts in paired combinations. Cohen’s kappa (*κ*) was used to assess inter-rater reliability. Disagreements were resolved through discussion with consultation from a third reviewer where necessary. This process was repeated until *κ* ≥ 0.75 for all reviewer pairs, indicating excellent agreement (13). The remaining titles and abstracts were screened by a single reviewer.

Eligible full-text articles were then assessed using the same process. The required *κ* value was reached after the third round of title and abstract screening and the first round of full-text screening.

### 2.3 Data extraction and quality assessment

A data extraction form was developed in Microsoft Excel 2021. For each included study, the following data were extracted: authors, year of publication, study design, setting, population, outbreak disease, outbreak phase, sample size, stigma measure, intervention, comparator, and outcomes.

Risk of bias was assessed using the revised Cochrane risk of bias tool (RoB 2) (14) for randomised controlled trials (RCTs), the Risk Of Bias In Non-randomized Studies of Intervention Version 2 (ROBINS-I V2) (15) for observational studies and the Critical Skills Appraisal Programme (CASP) Qualitative Studies Checklist (16) for qualitative studies.

## 3 Results

**Figure 1:**
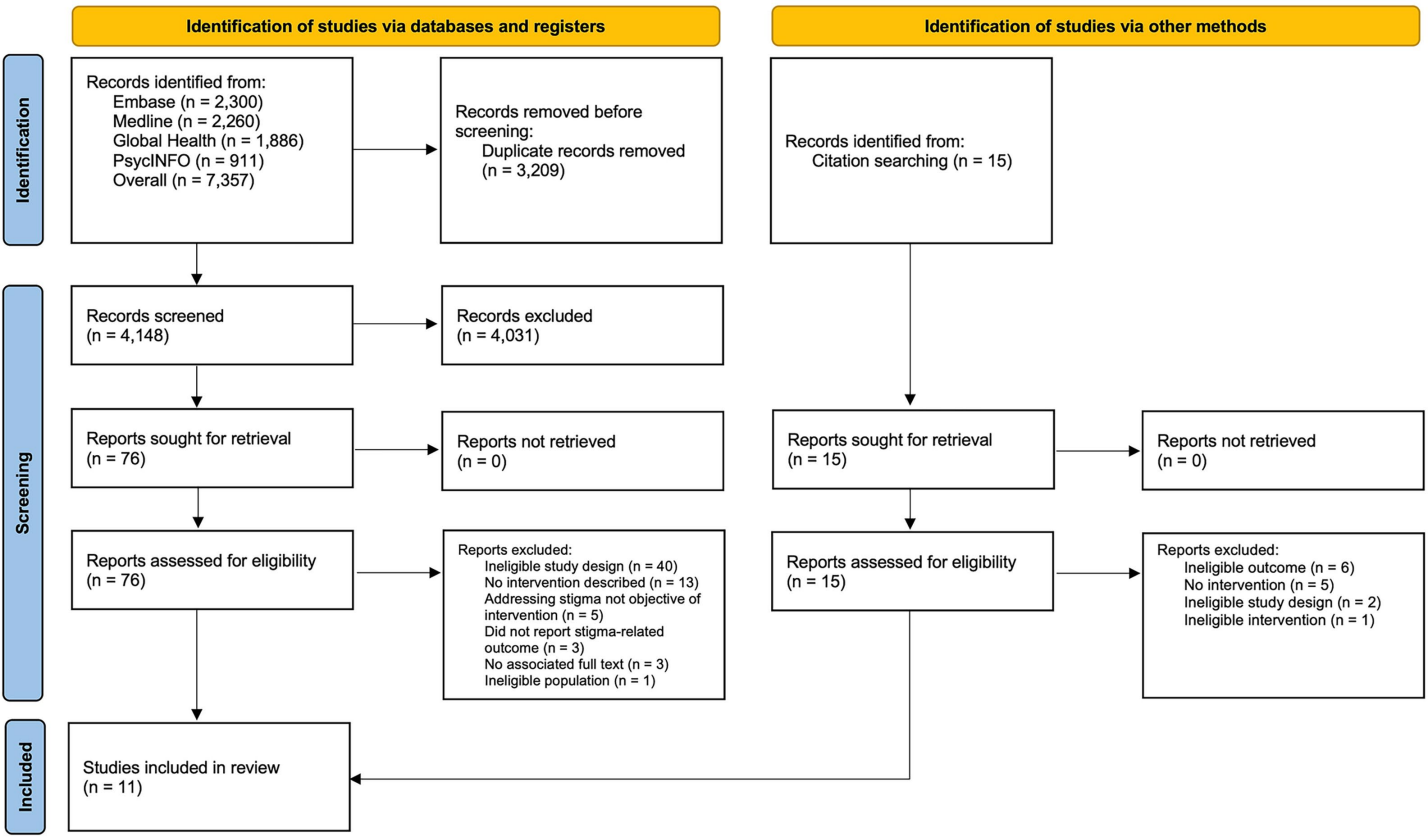
PRISMA flowchart summarizing study identification, screening, and selection.

The search identified 4,148 unique records, of which 76 were retrieved for full-text review (Figure 1). An additional 15 potentially relevant studies were identified through reference list screening, one of which fulfilled the eligibility criteria. In total, 11 studies describing and evaluating interventions to address stigma in the context of infectious disease outbreaks were included in the review.

### 3.1 Characteristics of the included studies

Studies were published between 2008 and 2024 and were conducted in eight countries across Africa, Asia, Europe, and North America (Table 2). Infectious disease outbreaks represented were COVID-19 (*n* = 6), Ebola disease (*n* = 2), mpox (*n* = 1), SARS (*n* = 1), and a hypothetical infectious disease (*n* = 1), across response (*n* = 7) and recovery (*n* = 4) outbreak phases. Study designs comprised RCTs (*n* = 7) and qualitative studies (*n* = 4). Among the seven studies that applied quantitative measures of stigma, three did not report evidence of scale validation.

**Table 2:** Characteristics of included studies.

1st author, year	Country, disease	Population	Sample size	Intervention	Stigma measure; any validation	Outcome; risk of bias
Randomised controlled trials						
Islam and Pakrashi, 2021 (17)	India, COVID-19	Adult residents	1,081 (intervention); 1,057 (control)	Phone-based delivery of accurate COVID-19 information and anti-stigma messaging	Two composite stigma measures; no	↓; some concerns for bias*
Lu et al., 2021 (26)	China, COVID-19	University students returning from Wuhan to non-Hubei provinces	31 (intervention); 32 (control)	Brief online “social support” reading and writing psychological exercise	Perceived Personal Discrimination Scale; yes	↓; some concerns for bias*
Smith, 2012 (19)	United States, Disease X (hypothetical)	Undergraduate students	333 (16 experimental conditions)	Exposure to different message frames in a hypothetical infectious-disease alert	Stigma-belief and restrictive-measures scales; yes	↑ (high-peril messaging); some concerns for bias*
Techapoonpon et al., 2023 (25)	Thailand, COVID-19	Recovered persons who had been admitted to hospital for COVID-19	71 (intervention); 71 (control)	Brief self-directed online programme on COVID-19 stigma and emotional first-aid techniques	Adapted version of COVID-19-related stigma questionnaire; yes	↓ (days 7 and 14); some concerns for bias*
Tidwell et al., 2024 (20)	Kenya, COVID-19	Residents of informal settlements	515 (intervention 1); 516 (intervention 2); 494 (control)	Behaviourally framed text messages emphasising (1) reciprocity or (2) community support	Survey of knowledge, attitudes and behavioural intentions; no	↔; some concerns for bias*
Valeri et al., 2021 (18)	United States, COVID-19	Adult residents	250 (control); 243 (intervention 1); 249 (intervention 2); 246 (intervention 3)	(1) Information sheet only; (2) plus video on social support; (3) plus video of recovered person sharing experience	Adapted HIV stigma scale; no	↓ (interventions 2 & 3); high risk of bias*
Wang et al., 2024 (27)	China, COVID-19	Male adults	70 (within-sample, before/after comparison)	24 IU intranasal oxytocin (based on social behaviour and empathy mechanism)	Stigma-judgement paradigm; yes	↔; low risk of bias*
Qualitative studies						
Biesty et al., 2024 (21)	United Kingdom, mpox	Outbreak responders and GBMSM	11 (key-informant interviews); 15 (workshops)	Community-led public-health campaigns and messaging through LGBTQ+ organisations to reduce stigma and increase vaccine uptake	Qualitative interviews / focus groups; N/A	Mixed results; largely methodologically sound †
Collier et al., 2023 (22)	Sierra Leone, Ebola disease	Affected community members / leaders	134 (total participants)	Community-driven sensitisation campaign using survivors, local leaders and health workers to disseminate trusted information	Qualitative interviews / focus groups; N/A	↓; methodologically sound †
Crea et al., 2022 (23)	Sierra Leone, Ebola disease	Affected community members / leaders	228 (total participants)	Community protection bylaws and education led by local leaders, health workers and NGO staff	Qualitative interviews / focus groups; N/A	Mixed results; mostly methodologically sound †
Siu, 2008 (24)	Hong Kong, SARS	Recovered persons	170 (observation only); 30 (in-depth interview)	Follow-up clinics for SARS survivors with separate entrances and elevators for privacy	Qualitative interviews; N/A	↑; methodologically sound †

↓Reduction in stigma; ↑ Increase in stigma; ↔ No clear change in stigma. *Assessed with Cochrane revised risk-of-bias tool for RCTs (RoB 2). †Assessed with CASP qualitative-studies checklist. IU, International units; GBMSM, Gay, bisexual and other men who have sex with men; NGO, Non-governmental organisation; SARS, Severe acute respiratory syndrome; UK, United Kingdom; US, United States.

### 3.2 Narrative synthesis

Evaluated interventions were grouped into four domains: knowledge exchange; community involvement and leadership; policy and service design; and psychosocial support (Table 3).

**Table 3:** Stigma reduction intervention thematic domains.

Domain
Knowledge exchange Public health communication, education, and community engagement.
Community involvement and leadership Activism by or with people with lived experience of the illness and affected community organisations, aiming to shift social norms and promote equity.
Policy and service design Structural or operational adaptations, such as changes to infrastructure or procedures intended to reduce stigma or prevent its institutional reinforcement.
Psychosocial support Psychological counselling, social services, or skill-building resources.

#### 3.2.1 Knowledge exchange

Four RCTs focused on knowledge-exchange interventions. In India, a telephone service delivering accurate COVID-19 information and government guidance on avoiding stigma led to lower composite stigma scores (towards people with COVID-19, frontline workers, and select marginalised groups) relative to the control group (17).

In the United States (US), a trial comparing three versions of a brief online resource found that adding a short video of a COVID-19 survivor describing their experience led to greater reductions in stigma perceptions than an information sheet alone, with outcomes measured immediately after the intervention (18). A vignette-based experiment among US undergraduates examined a hypothetical infectious disease alert. Messages that emphasised high peril (for example, severe symptoms and fatality) increased perceptions of dangerousness and fear, which were associated with stronger stigma beliefs and greater support for restrictive measures, compared with less alarmist framing (19).

In Kenya, a large RCT tested behaviourally framed text messages encouraging supportive attitudes towards people with COVID-19. The control message explained that anyone can get coronavirus and that those infected should stay isolated until recovered, while emphasising that they should still be “loved, cared for and accepted by friends and neighbours.” (20) Two additional versions added either a reciprocity cue (“Treat others with coronavirus how you would like to be treated”) or a social-benefit cue (“Supporting one another will help our community and nation through this difficult time”). Neither alternative message reduced stigma or increased caregiving intent compared with the control, which already contained explicit anti-stigma content (20).

In addition to these more formal communication interventions, several of the community-led approaches described in the following section also incorporated knowledge exchange activities, often delivered by people with lived experience or trusted local actors (21–23). These approaches were generally perceived as helpful in correcting misinformation, reducing fear, and supporting reintegration, but participants also raised concerns about over-reliance on community and third-sector organisations for health education and stigma mitigation (21–23).

#### 3.2.2 Community involvement and leadership

Three qualitative studies examined the impact of community involvement and leadership on outbreak-related stigma.

Two of these studies were conducted in Sierra Leone during the West African Ebola disease outbreak. In the first, key informant interviews and focus groups with affected community members highlighted the role of outbreak response measures such as physical distancing in exacerbating stigma. Community-led reintegration efforts, education initiatives, and protection bylaws were perceived to reduce gossip, fear, and overt discrimination (23).

The second study from Sierra Leone study explored Ebola disease-related knowledge, beliefs, and trusted sources of health information among affected communities. Participants reported that community-driven efforts, such as peer education by survivors, visible involvement of local leaders, and sensitisation by community health workers, supported more accepting attitudes and facilitated survivor reintegration into social and economic life (22).

The third study examined the 2022–2023 mpox outbreak in the UK through interviews and participatory workshops with gay, bisexual, queer, and other men who have sex with men, alongside other stakeholders (21). Participants described anticipated and experienced stigma arising from public health and media messaging that framed mpox as concentrated among gay and bisexual men, including fears of being blamed, judged, or labelled as irresponsible. Community-based LGBTQ+ organisations and sexual health services were reported to play a central role in rapidly organising peer-led campaigns, designing, and delivering tailored information, countering harmful narratives, and supporting non-stigmatising engagement with vaccination and care. At the same time, some participants expressed concern that these organisations were expected to carry much of the communication and outreach effort during the outbreak, highlighting uncertainties about sustainability and the need for more consistent institutional support.

#### 3.2.3 Policy and service design

One ethnographic study examined how structural and service-level adaptations shaped stigma following the SARS outbreak in Hong Kong (24). Dedicated outpatient clinics were established for people who had recovered from SARS, with separate entrances, lifts and waiting areas. Although intended to provide privacy and reassurance, survivors reported that these arrangements marked them out as different and implied that they remained infectious or “dirty.” Some respondents described how these experiences discouraged them from continuing follow-up care or seeking formal health services in future outbreaks (24).

#### 3.2.4 Psychosocial support

Two RCTs evaluated psychosocial support interventions in the context of COVID-19. A study in Thailand examined a brief, self-directed online destigmatisation programme delivered via interactive video to individuals 1–2 days before discharge from hospital after COVID-19. The programme combined psychoeducation about COVID-19-related stigma, its mental health impacts, and behaviours to minimise stigma with simple emotional first aid and coping techniques. Stigma scores declined more in the intervention group than in the control group at 7 and 14 days after discharge, although between-group differences were no longer evident by day 28, suggesting a short-term effect that diminished over time (25).

Similarly, another RCT investigated the effect of an online intervention designed to enhance perceived social support in 63 college students who had returned home from Wuhan, China after the first wave of COVID-19. Participants read brief accounts describing supportive interactions during the outbreak and then completed a reflective writing exercise about support in their own lives. Those who completed the task reported increased perceived social support and decreased perceived discrimination compared to those in the control condition when assessed immediately post-intervention (26).

A final study was an outlier that did not fit neatly within these thematic domains. This double-blind, placebo-controlled experimental study in China examined whether intranasal oxytocin could modulate explicit and implicit COVID-19-related stigma judgements, drawing on prior evidence that oxytocin can influence social bonding, anxiety, and social evaluation. Among 70 male participants, oxytocin did not reduce stigma overall. Instead, the authors reported that oxytocin appeared to amplify differences by social anxiety level: in individuals with low social anxiety, oxytocin reduced personal blame attributed to people associated with COVID-19, whereas among those with high social anxiety it heightened blame (27).

### 3.3 Quality assessment

Most included RCTs (5/7, 71%) were adjudicated to have some concerns for risk of bias; one was high risk and one low risk. All four qualitative studies were mostly methodologically sound (Table 2).

## 4 Discussion

This review synthesises the current evidence on stigma reduction interventions in the context of infectious disease outbreaks. Despite searching across multiple databases and outbreak-prone diseases, only 11 eligible studies were identified. The scarcity of evaluated interventions in this area is striking given the prominence of stigma in outbreak narratives and guidance. This underscores the need for more systematic attention to how stigma is addressed in preparedness, response, and recovery. While limited, the existing evidence suggests a few promising avenues for stigma mitigation, alongside examples of interventions that had little effect or risked exacerbating stigma.

Among the interventions identified in this review, the strongest evidence of effectiveness was for those embedding anti-stigma messaging alongside health information (17, 18). This aligns with broader literature on infectious disease communication, which emphasises that factual knowledge alone is often insufficient to shift deeply rooted social attitudes and fears (3, 7). In contemporary outbreak settings, where misinformation can spread rapidly through digital and social media channels, there is a critical need for stigma-sensitive messaging delivered by trusted sources (28). However, framing matters: the finding that high-peril messaging may increase stigma illustrates how communication strategies can produce unintended effects when content and tone are not carefully considered (19).

Community involvement and leadership also emerged as an important domain for stigma reduction. Across the qualitative studies, community-led reintegration efforts, educational activities and communication were described as helping to address fear and support acceptance (21–23). These approaches also reflect a broader ethical imperative: outbreak responses should be shaped by, and not only for, affected communities. Such involvement can improve relevance and trust, while reducing the likelihood that response measures unintentionally compound stigma.

All three studies on community involvement included education efforts led by individuals with lived experience. Similarly, the RCT conducted by Valeri et al. (18) featured a video of a person recovered from COVID-19 sharing their experience. In the broader stigma literature, social contact refers to interaction between people with and without a condition and is considered one of the most effective stigma reduction strategies (29–31). The term ‘social contact’ is not commonly used in outbreak stigma reduction contexts, where language associated with infection prevention, such as ‘social distancing’, may complicate interpretation. However, the findings demonstrate how forms of social contact can still be incorporated safely in outbreak contexts, for example through digital platforms or by involving recovered individuals. Such approaches can help foster empathy, challenge stereotypes, and reduce perceived divisions between affected groups and the wider public.

The results also highlight how well-intentioned interventions aimed at reducing stigma may inadvertently exacerbate it. Structural adaptations, such as specially designated services or entrances intended to protect privacy, may reinforce perceptions of “otherness” or ongoing risk and can discourage care-seeking (24). Careful attention to stigma risk in designing and communicating outbreak response and recovery measures is therefore essential. Conceptual frameworks such as the Health Stigma and Discrimination Framework (3) and the hourglass model (9) can support more systematic consideration of how policies, services and communication strategies may influence stigma across the course of an outbreak, and help anticipate and minimise unintended harms.

The review also identified brief psychosocial interventions that yielded rapid reductions in internalised and perceived stigma (25, 26). These findings mirror evidence from the HIV and mental health literature where interventions like peer-led counselling, narrative-based approaches and cognitive reframing have been shown to effectively reduce stigma (32, 33). However, as demonstrated by Techapoonpon et al. (25), the effectiveness of these single-session intervention tends to wane with time when there is no reinforcement or integration into ongoing support. This highlights the need for stigma-reduction interventions designed to achieve durable, long-term impact and for studies to have sufficient follow up to allow for longer-term outcome assessment.

Several features of the available studies limit how confidently their findings can be applied. Quantitative evaluations were generally small, drawn from single contexts, and characterised by short follow-up periods. Outcomes were mostly based on self-completed questionnaires that are vulnerable to social desirability and related response biases. The qualitative studies provided essential insight into context, acceptability and process but did not offer detailed effectiveness data or compare approaches. Taken together, these features mean that the benefits observed in individual studies should be interpreted cautiously.

Heterogeneity in how stigma was defined and measured adds further uncertainty. Several evaluations relied on bespoke measures without psychometric validation or explicit theoretical grounding. This affects confidence in baseline estimates and observed changes. The absence of standardised measures also limits meaningful comparison across studies, restricting the ability to synthesise findings or assess the consistency and magnitude of intervention effects. An earlier review of infectious-disease stigma scales highlighted similar concerns, including lack of robust development processes and missed opportunities to use consistent assessment tools (11). Cross-outbreak instruments, such as the (Re)-emerging And ePidemic Infectious Diseases (RAPID) stigma scales, offer one potential route towards more consistent measurement across diseases and settings, though context-specific piloting remains important (34).

The review has several additional limitations. The included studies were unevenly distributed (six on COVID-19 versus one on mpox), and predominantly from high- and middle-income settings. The paucity of evidence from low-income settings is unlikely to reflect a lack of stigma relevance in these contexts, but rather structural inequities in global research production and evaluation capacity. This restricts the generalisability of our findings to other outbreak disease contexts and to low-resource regions underrepresented in published literature. Finally, most of the included RCTs were assessed to have ‘some concern’ or high risk of bias. This further tempers our confidence in the reported effectiveness of the included interventions.

The weakness of the intervention evidence base is particularly concerning given the pervasiveness of stigma in outbreaks, and the potential for response measures to cause further social division. Building a stronger evidence base will require study designs that can generate insight into both effectiveness outcomes and the processes through which they are achieved. Important next steps include pragmatic trial approaches, such as stepped-wedge cluster RCTs, which can help assess effectiveness under real-world conditions. In addition, qualitative and mixed-methods implementation research is essential not only to document intervention effects, but also to elucidate underlying mechanisms, contextual influences, and the risk of unintended or counterproductive effects, including those arising from well-intentioned outbreak response measures. Until more robust evidence is available, practice may need to be steered by expert consensus guidance, with careful adaptation of evidence from non-outbreak contexts.

Recent guidance illustrates how stigma mitigation can be operationalised in practice despite current evidence gaps. For example, the International Severe Acute Respiratory and Emerging Infection Consortium (ISARIC) Anti-Stigma Guidelines provide cross-outbreak recommendations for integrating stigma-sensitive approaches into risk communication plans, outbreak preparedness frameworks, and health worker training, including guidance on stigma-sensitive communication, meaningful community engagement, and the design and delivery of respectful outbreak services (35). Such frameworks demonstrate how insights from qualitative and quantitative evidence can be translated into concrete actions across preparedness, response, and recovery phases, rather than implemented as isolated or *ad hoc* interventions.

The evidence base for stigma-reduction interventions in outbreaks remains limited, offering tentative indications of what may be effective. Nonetheless, several approaches appear promising, including stigma-sensitive risk communication, meaningful community involvement, psychosocial support, and increasing attention to how policies and services may inadvertently reinforce otherness. Strengthening this evidence will require rigorous, contextually-informed evaluation. At the same time, policymakers and outbreak responders can integrate the proposed approaches into preparedness, response, and recovery efforts as practical steps to mitigate stigma, while more robust evidence continues to develop.

## Data Availability

The original contributions presented in the study are included in the article/supplementary material, further inquiries can be directed to the corresponding author/s.
